# Social media as a workplace panopticon: The development and validation of social media monitoring by workplace contacts scale

**DOI:** 10.1371/journal.pone.0319429

**Published:** 2025-03-19

**Authors:** Hamnah Rahat, Sadia Nadeem

**Affiliations:** FAST School of Management, National University of Computer and Emerging Sciences, Islamabad, Pakistan; Southeast University, CHINA

## Abstract

The monitoring of employees’ private social network accounts by employers and colleagues has become increasingly prevalent, yet research in this area remains limited. To address this gap, the present study developed and validated a scale to measure social media monitoring by workplace contacts (SMMWC). The scale, comprising fifteen items, was developed using Hinkin’s (1998) approach to scale development and has four dimensions based on the concept of panoptic effect by Foucault (1977) and Botan (1996). While Study 1, based on 334 employees, focused on scale development, Study 2, based on 302 employees, replicated the factor structure of the SMMWC scale and examined its impact on outcomes, using a time-lagged design. The SMMWC scale demonstrated strong psychometric properties, including factorial validity; discriminant validity with electronic performance monitoring and user perceptions of social media monitoring; and criterion-related validity with online disclosure, social capital, emotional exhaustion, and self-concept clarity. Notably, SMMWC was positively associated with online disclosure in both the studies and was significantly related to emotional exhaustion and self-concept clarity in Study 2, suggesting that SMMWC can influence employees’ online behavior and psychological well-being.

## Introduction

The evolution of information technology and a transformative shift in work paradigms within the realm of cyberspace have turned employee performance monitoring (EPM) into a panopticon-like environment. Similar to Jeremy Bentham’s theoretical prison design, where inmates felt constantly observed, electronic performance monitoring (EPM) devices, encompassing technologies such as video monitoring, call monitoring, biometric verification, and scrutiny of email and internet usage [1], have created an atmosphere of continuous observation and self-vigilance among employees within the workplace. Simultaneously, over the past decade, social media has introduced an additional layer of surveillance, where employees perceive monitoring of their social media activities from their organization and colleagues, further exacerbating privacy concerns and ethical dilemmas [2,3]. While traditional EPM is confined to workplace boundaries and its impacts on employee and organizational outcomes are well-documented, social media monitoring extends beyond the workplace, representing an emerging and less understood area of influence.

Many individuals report feeling watched and monitored on social media [4–6]. In the professional setting, employers are increasingly using social media for screening potential job candidates and monitoring the activities of existing employees [7–9]. This practice has even resulted in documented legal cases where employees faced termination due to their social media posts [10]. Furthermore, some studies reveal employees’ discomfort in deciding whether to accept or reject friend requests from colleagues and supervisors [11–13]. This discomfort is frequently driven by the perception of being under social media surveillance, which heightens anxiety about how online behavior might be interpreted in the workplace [11–13]. Internal pressures, such as the need to maintain workplace relationships, often lead employees to accept such requests. This dynamic reflects broader concerns about the blurring of boundaries between personal and professional spaces in the digital age where employees may feel pressure to curate their online presence to meet workplace expectations [14].

Despite the prevalence of these issues, the existing literature on social media monitoring by workplace contacts is limited by several key factors. First, there is a lack of a clear definition of social media monitoring within workplace relationships. Most studies focus on broader concepts of social media surveillance (e.g., [4,15]), online information seeking (e.g., [16–24]), or online privacy concerns (e.g., [25–30]), failing to capture the unique dynamics of perceived social media monitoring from employers and colleagues. This study defines Social Media Monitoring by Workplace Contacts (SMMWC) as the perception or reality of surveillance that employees experience from their workplace contacts on social media platforms. Workplace contacts include individuals from higher hierarchical levels (e.g., employers and supervisors), the same hierarchical level (e.g., colleagues), or lower levels (e.g., subordinates). SMMWC encompasses behaviors and mechanisms through which workplace contacts observe, assess, or act upon an employee’s social media activity.

Second, the available measurement tools (e.g., [31,32]) highlight various dimensions of online monitoring but are limited in their applicability to workplace contexts. Table 1 presents these instruments. Scales such as the Facebook Surveillance Scale [24] and the Facebook Connection Strategies Scale [23] assess behaviors like social monitoring by friends or strangers but do not address monitoring within professional relationships. These scales assess a spectrum of behaviors related to using Facebook for social monitoring, ranging from benign activities such as connecting and staying updated on friends [23,24] to more intrusive actions like spying and gathering confidential information about others through social networking sites [24].

**Table 1:** Instruments measuring surveillance on SNS.

Measure name and reference	Perpetrator or Victim	Type of Information Seeker	Number of Items	Psychometric Tests on the Instrument
Facebook surveillance scale by Stiff (2019)	Perpetrator	Friend, latent ties, and stranger	14	EFA, CFA and Regression Analyses
Facebook connection strategies scale by Ellison et al. (2011)	Perpetrator	Close friends, latent ties, and strangers	13	EFA and Regression Analyses
Facebook information-seeking scale by Tong (2013)	Perpetrator	Ex-romantic partner	17	EFA and Regression Analyses
Electronic intrusion perpetration scale by Reed et al., (2016)	Perpetrator and Victim	Romantic partner	6	T-Tests and Regression Analyses
Cyber aggression in relationships scale by Watkins et al. (2018).	Perpetrator and Victim	Romantic partner	17	EFA, CFA, and Correlations
Online obsessive relational intrusion scale by Chaulk and Jones (2011).	Perpetrator	Relational Intrusor	38	EFA
Facebook survey by Lyndon et al. (2011).	Perpetrator	Ex-romantic partner	13	EFA, Frequencies comparison with the related instrument
Facebook partner monitoring by Darvell et al. (2011).	Perpetrator	Romantic partner	17	MANOVA and regression analyses
Cyberdating violence inventory scale by Morelli et al. (2018).	Perpetrator and Victim	Romantic partner	22	EFA, CFA, and correlations with related instruments
Employer use of SNS by Hurrell et al. (2017).	Victim	Current and past employees	7	Regression analyses
Employer use of SNS monitoring by Suen (2018).	Victim	Current employees	3	EFA and regression analyses

Similarly, scales designed to measure social media monitoring in personal relationships, such as romantic partner or stalking behaviors [16–22], are less applicable in the workplace context due to differences in dynamics and intentions. These scales include items ranging from checking a person’s profile and activities to sending provocative or threatening messages, sharing private or embarrassing information without permission, pressuring for intimate information, insulting, and spreading rumors, which differ from the power dynamics and professional repercussions inherent in workplace monitoring. The challenge in adapting monitoring scales designed for friends and romantic interests lies in their lack of specificity when applied to the workplace context. Also, these scales primarily focus on individuals who engage in monitoring others on social media, rather than addressing the experiences of those who may be the subjects or victims of such monitoring.

Moreover, existing scales on privacy concerns in social media are also insufficient for measuring the social media monitoring that employees may perceive from their professional contacts. Many privacy scales focus on users’ concerns related to online platform vendors, such as the Internet privacy concern scales by Malhotra et al. [25] and Hong and Thong [26] and social media privacy concerns scales by Koohang et al. [27] and Rehman et al. [28]. Additional scales that exist measure interactions with peers on the Internet, such as the Peer Privacy Concern Scales by Zhang et al. [29] and Ozdemir et al. [30]. While these scales demonstrate that users do have privacy concerns online, they fail to account for the specific context of monitoring experienced by employees from their professional contacts. Privacy scales usually emphasize control over personal information and the risks of exposure to a broader, often anonymous audience. In contrast, social media monitoring by professional contacts introduces unique challenges, including power dynamics and potential professional repercussions.

While scales such as Hurrell et al.’s [31] and Suen et al.’s [32] address employer use of SNS, they do not account for the broader context of social media monitoring from workplace contacts. Hurrell et al. assessed employer use of SNSs through activities, including recruitment, screening, communication, collaboration at work, discipline, assessing performance, and encouraging social events outside of work [31]. Suen et al.’s scale included items such as being asked to connect with the employer on SNS, having one’s profile screened on SNS, and using SNS to make decisions [32]. However, these scales primarily concentrate on the employer’s utilization of SNS and disregard several vital factors, currently trending in the literature, that influence the perception of being monitored on social media such as the influence of colleagues who follow employees’ social media activities [11–13], the organization’s social media policies, past employer responses to employee social media posts, and employees’ social media privacy settings.

Third, research in this area is sparse. While EPM has been widely studied [1], it primarily focuses on workplace-specific surveillance, such as biometric tracking, video monitoring, and email scrutiny [1]. In contrast, social media monitoring extends beyond workplace boundaries [32], introducing an omnipresent form of surveillance. Despite its prevalence, its effects on employee behavior, relationships, and organizational outcomes are underexplored. This gap underscores the need for empirical research and a robust measurement tool to address the unique aspects of workplace social media monitoring.

Given these gaps in the existing literature, this study aimed to develop and validate a scale for measuring Social Media Monitoring from Workplace Contacts (SMMWC) following Hinkin’s (1998) scale development procedure [33]. To deepen our understanding of the measure, this study draws on Foucault’s concept of panopticon [34], as well as Botan’s work on the panoptic effect [35]. Building on the four elements of the panoptic effect of EPM devices as suggested by Botan [35] this study introduces the SMMWC scale, with Perception, Potential, Management Policy, and Maturity as its key dimensions.

Unlike broader social media surveillance scales or privacy concern scales, the SMMWC scale targets the specific context of social media monitoring within workplace relationships. It goes beyond scales measuring employer utilization of SNS. By using a panoptic lens, the scale offers deeper and more holistic insights into how employees perceive and experience this type of surveillance. The scale not only measures whether employees feel they are being surveilled on social media, but also their perceptions of the potential for surveillance, the management policies regarding social media use, and the maturity or effective integration of these management policies. By considering these additional dimensions, the SMMWC scale offers a comprehensive understanding of the panoptic effect of social media surveillance in the workplace, thereby offering deeper insights into its implications for both employee and organizational outcomes.

The absence of a dedicated scale has led to a scarcity of studies examining the impact of social media monitoring by employers and colleagues on various employee and organizational outcomes. However, substantial evidence links privacy concerns to online disclosure, [36–38]). Additionally, research has shown links between social media usage and self-concept clarity [39], as well as employee stress and well-being in the contexts of electronic performance monitoring [40–42] and social media research [43–45]. Thus, following the development of the SMMWC scale, this study also examines the impact of SMMWC on various employee outcomes.

This research makes a significant contribution to the body of knowledge by examining the impact of SMMWC on various employee outcomes. Practitioners and organizations can benefit from this research by utilizing the findings from this study and carrying out future research using the SMMWC scale, to make informed decisions and to effectively implement appropriate social media policies and guidelines.

## Literature review

While the original panopticon by Bentham was a watch tower in prison, Foucault’s panopticon described a society of surveillance where the idea of being watched impacts the actions one would perform [34,46]. The concept of panopticon has been applied to EPM in organizations where high-security towers with guards monitoring prisoners were replaced by the use of electronic monitoring devices such as cameras, biometric devices, GPS, and finger scanners [35]. Many researchers have theorized that SNS function as a modern panopticon (for example, [47–51]), creating a perceived sense of surveillance over users that controls users’ behaviors even prior to norm violation. Botan [35] suggested that, for the panoptic effect to work for EPM devices, there should be an interaction of four elements: 1) employee perception of being surveilled, 2) surveillance potential of the technology, 3) management policy, and 4) maturation (pg. 299). Considering that SNS impact users’ online behavior through the panoptic effect, the concept of the panopticon and the dimensions of the panoptic effect by Botan [35] were used as a starting point to develop the dimensions and items for the SMMWC scale.

### Dimensions of SMMWC

Building on Botan’s dimensions of the panoptic effect, the key dimensions of Social Media Monitoring and Workplace Control (SMMWC) include employees’ perception of being under social media surveillance (Perception), the surveillance potential of social networking sites (Potential), organizational policy regarding social media surveillance (Management Policy), and the level of maturity in social media surveillance (Maturity).

#### Perception: Employees’ perception that their social media is under surveillance.

The main element of the original panopticon was the perception of the prisoners that they were being watched from the centralized guard tower in the prison without knowing exactly when they were being watched [46]. By applying this concept to electronic performance monitoring (EPM) and social media surveillance, it becomes evident that the mere belief that management is overseeing employees’ performance, or online activities can influence their conduct. Even if employees are not being monitored and are not aware of any monitoring, the suspicion of surveillance can produce a panoptic effect [52].

#### Potential: Surveillance potential of SNSs.

The second element of the panoptic effect is the surveillance potential. According to Botan [35], for electronic surveillance technology to maintain its potential, it needs to have four characteristics, including a degree of visibility, a degree of invisibility, a degree of record production, and a degree of technologically driven data analysis. Extending the concept to EPM, where EPM devices, such as video cameras, emails, phone call monitoring, etc., make the employee behavior and performance visible to the surveillance authority while keeping the authority “invisible”, i.e., managers are not physically present to observe the employees but can observe the employees through the devices [53]. Sophisticated technologies also allow managers to produce ample data and complex data analyses [1], thereby exceeding the surveillance potential of the EPM as compared to panoptic prisons. However, unlike panoptic prisons or electronic panopticons where surveillance is imposed on prisoners and employees, social media is an omniopticon or “participatory panopticism” where users voluntarily participate and open their lives for monitoring by the audience [50]. Users render information about themselves visible to the audience, while the audience (if permitted by privacy settings) can observe this information while remaining invisible to the users [50]. The unlimited storage capacity and search engines on social networking sites (SNSs) enable audiences to swiftly retrieve old records about users if the users have not maintained appropriate privacy settings.

#### Management policy: Management policy regarding the use of social media surveillance.

In Foucault’s [34] discussion of Bentham’s prison, it was the fear of consequences that reduced crime by initiating a process of thinking, preventing prisoners from performing the actions they were forbidden to engage in. Applying these principles to EPM devices, management policies can determine when EPM technology can be used for surveillance and how the results can be used to ensure quality and for disciplinary purposes [35]. For social media monitoring, management policies are social media-related policies that legalize taking actions against an employee based on information shared on the employee’s social media account. Even if there are no policies in writing, employers’ use of social media information for employee career-related decisions such as hiring, promotion, and firing (labeled as cyber-vetting [54,55]) a part of the company’s policy or standard way of working may result in employees’ perception of being monitored.

#### Maturity: Maturity of social media surveillance.

The fourth and last element of the panoptic effect is maturation, which refers to how effectively surveillance technology is integrated with management policies [35]. This is the integration of the first three factors that work together to increase the panoptic environment. Maturity in surveillance technology is achieved when surveillance procedures are well established, employee and legal opposition are resolved, and the results of monitoring are a part of organizational decision-making and disciplinary proceedings [35]. However, achieving maturity in social media surveillance may be challenging due to varying privacy and employer protection laws across states and countries [56]. This could pose difficulties for employers in establishing social media monitoring procedures and resolving legal and employee issues. Given the history of cases where employees have been fired based on their social media information [57], the maturation of social media surveillance by employers can occur when social media monitoring becomes an integral part of organizational decision-making and disciplinary procedures, particularly if there are social media policies in place. Consequently, employees are likely to perceive monitoring from workplace contacts when the organization or colleagues have taken action against employees for their activities on social media, especially when these activities violate organizational social media policies or implied social media norms.

### SMMWC in the nomological network

To develop a new scale, it is important to explain where it fits within the nomological network [33]. This involves administrating the new measures in conjunction with established ones to explore the relationship between established measures and developed constructs [33]. In the case of SMMWC, there are two interconnected yet distinct concepts: traditional electronic performance monitoring (EPM) and users’ perception of social media monitoring from all audiences.

EPM can be defined as “the now-common use of technological means to observe, record, and analyze information that directly or indirectly relates to employee job performance” [1]. These monitoring methods can include a range of devices such as cameras, biometric devices, GPS, and finger scanners. While monitoring through EPM and SMMWC, both forms of monitoring involve the use of technology to observe and record information, they differ in their scope and purpose. EPM primarily operates within the physical domain of the workplace during employees’ work hours. It is often aimed at enhancing performance, preventing loss, employee development, administrative purposes, and ensuring the safety of employees [1]. In contrast, SMMWC extends beyond the physical workplace and involves monitoring employees’ social media accounts, an activity that is not directly related to their job performance. Thus, the primary purpose of social media monitoring is often associated with surveillance and control over employees.

Another construct related to SMMWC is users’ perception of social media monitoring from all audiences, which is referred to as “social media monitoring (SMM)”, in this study, while social media monitoring from workplace contacts is referred to as SMMWC. Users may perceive social media monitoring from audiences and existing literature has scales measuring social media monitoring from different audiences such as friends [23,24], families [58], and romantic partners [16–18,21]; however, these scales measure perpetration rather than the victimization of social media monitoring from multiple audiences. Nevertheless, researchers have introduced and measured phenomena such as users’ concerns about mediated lurking, which measures apprehensions users have regarding others monitoring their social networking sites [59] and privacy concerns on SNS (for example, [36–38]). Considering that users may have privacy concerns regarding SNS because of social media monitoring, scales on privacy concerns can be used to tap the phenomena. SMM and SMMWC are related constructs because both can impact users’ decisions on disclosing personal information online, via SNS, due to the presence of monitoring. However, social media monitoring by workplace contacts can potentially affect employees’ professional outcomes, such as career advancement and disciplinary decisions, users may exercise more caution when disclosing information on their social media accounts if they perceive monitoring from their workplace contacts compared to other audiences. Based on this understanding, it is hypothesized that:

Hypothesis 1: Social Media Monitoring from Workplace Contacts (SMMWC) is a distinct construct from electronic performance monitoring (EPM) and social media monitoring from all contacts (SMM).

### Impact of the nomological network on outcomes

A developed scale should establish criterion validity by hypothesizing and testing how a developed variable predicts other variables [33]. While little is known about how the new variable SMMWC will impact different outcomes, existing outcome variables studied with EPM, and social media monitoring can be used to predict the impact of SMMWC. Thus, based on SMM and EPM literature, which is discussed in this section, SMMWC was anticipated to predict employees’ job satisfaction, invasion of privacy, online disclosure, identity clarity, and emotional exhaustion. It was also expected to predict social capital.

Past literature has found that monitoring through EPM devices is negatively associated with job satisfaction [60,61] and organizational commitment [60]; and positively associated with invasion of privacy [62,63]. Furthermore, studies on employer use of SNS to screen or monitor employees found that employer use of social media for screening/monitoring positively affected perceived invasion of privacy [64] and perceived privacy violation [32]. Considering that SMMWC is not directly related to employees’ performance and can be used as a surveillance tool by the organization, without any rationale linking it to employee performance, it is hypothesized that SMMWC will be negatively associated with job satisfaction and positively associated with invasion of privacy. The hypotheses are:

Hypothesis 2: SMMWC is positively associated with invasion of privacy.

Hypothesis 3: SMMWC is negatively associated with job satisfaction.

Similarly, it is expected that there will be similarities between the outcomes associated with SMM and SMMWC. Perhaps, the most immediate impact of SMM is users’ online disclosure on SNS. While research has explored the relationship between users’ concern for privacy and online disclosure [65–69], the existing literature has contradictory views on how users’ concerns about privacy impact how they disclose information. One explanation is that users’ concern for privacy should be negatively related to online disclosure as those who are keen to protect their private information are likely to disclose less information to prevent exposure. Although this relationship is supported by a stream of research [65–67], another stream of the literature suggests a “privacy paradox” where users disclose online information about themselves, despite privacy concerns [68,69]. Although the impact of SMMWC on employees’ online disclosure has not been directly measured, it is reasonable to assume a negative association between SMMWC and online disclosure. This is because employees may perceive non-work-related monitoring on social media, leading to a reduced inclination to disclose personal information online, to protect their privacy. Based on these considerations, the following hypothesis is proposed:

Hypothesis 4: SMMWC is negatively associated with online disclosure.

In addition to online disclosure of users on their SNS, changes in users’ identity due to SNS is another important area in social media research, where social media usage [39] and users’ online disclosure are associated with users’ self-concept clarity [70,71]. Self-concept clarity refers to the extent to which an individual possesses a clear, confident, and well-defined understanding of themselves [72]. SNSs provide platforms for identity disclosure, negotiation, and the experience of multiple identities [14]. According to the fragmentation hypothesis, exposure to different audiences on social media can hinder the development of an integrated sense of self [73–75]. Considering the pressure of monitoring by various audiences, it is plausible to perceive that SMMWC may lead employees to present a crafted identity online to impress their workplace audience, potentially resulting in decreased self-concept clarity. The hypothesis is as:

Hypothesis 5: SMMWC is negatively associated with self-concept clarity.

Employee stress and well-being are widely studied in EPM [40–42] and social media research [43–45] where multiple mechanisms such as the overwhelming demands of social media [76,77], cyberbullying victimization [78], etc. can contribute to user stress. It is perceived that SMMWC may lead to emotional exhaustion in employees, where emotional exhaustion refers to the feeling of being emotionally exhausted and cynic towards one’s work due to stress at work [79]. Employees may experience stress in several ways. Due to SMMWC, the disclosure of information on employees’ personal social media accounts can serve as a basis for employee hiring, promotion, and disciplinary relationships, and can also impact employee likability and respect in the workplace context [14]. The constant fear of deviating from a professional image and how workplace contacts perceive online disclosure can cause stress for employees. Therefore, the following hypothesis is proposed:

Hypothesis 6: SMMWC is positively associated with emotional exhaustion.

Despite numerous perceived drawbacks of SMMWC, one benefit of SMMWC can be improved relationships among workplace contacts. When employees perceive that workplace contacts follow and monitor their social media activities, it signals a desire to develop relationships beyond the workplace. This indicates a positive intention for relationship building among employees. Informational exchanges on social networking accounts provide opportunities for colleagues to engage, offer support, strengthen existing offline ties, and substantially reinforce long-term relationships with them [80]. Sharing personal experiences fosters social support and enhances friendships, contributing to employees’ social capital. Researchers have established SNSs as an important medium for developing social capital [23,81–83], and the percentage of coworker connections has been shown to affect online bridging social capital [84]. It is expected that perceiving that colleagues and employers are following employees’ social media activities may lead to increased social capital. Therefore, it is hypothesized that:

Hypothesis 7: SMMWC is positively associated with social capital.

## Methodology and results

This article is based on Study 1 and Study 2, following the guidelines outlined by Hinkin [33] for the development of the SMMWC scale. In Study 1, all necessary procedures were meticulously carried out to develop the SMMWC scale. This involved item generation, administering the questionnaire to participants, applying initial item reduction techniques to refine the scale, conducting confirmatory factor analysis to assess the underlying factor structure, and evaluating convergent and discriminant validity to examine the scale’s relationships with other constructs, as recommended by Hinkin [33].

To further validate the factor structure and explore the practical implications of the SMMWC scale, Study 2 was conducted. The primary objective of this study was to replicate the factor structure identified in Study 1, thereby confirming the stability and consistency of the scale across different samples, as advised by Hinkin [33]. Additionally, hypothesis testing was conducted to investigate the impact of SMMWC on various employee-related outcomes.

### Study 1

#### Dimensions and items generation.

The development of dimensions and items for the scale in this study employed both deductive and inductive approaches. A deductive approach was utilized, guided by theoretical foundations and definitions, specifically drawing from Foucault’s [34] and Botan’s [35] theoretical frameworks, as discussed. These frameworks informed the creation of dimensions for the SMMWC scale.

The first dimension, “Perception,” encompasses items that assess employees’ perception of being monitored on social media by their employers, supervisors, and colleagues. The second dimension, “Potential,” focuses on the surveillance potential of social networking sites (SNS) and includes items that reflect employees’ beliefs that their social media accounts can be easily monitored by their employers and colleagues. The third dimension, “Management Policy,” incorporates items related to organizational policies and practices regarding social media surveillance. Lastly, the fourth dimension, “Maturity,” pertains to the level of maturity in social media surveillance and includes items indicating instances where organizations or colleagues have acted against employees based on their social media activities.

In the initial phase of item development, a total of twenty-eight items were created for the scale. The process involved a combination of adapting pre-existing scales and generating original items through brainstorming and analyzing trends in the literature, following Zickar’s [85] guidelines for item writing, in order to encompass the identified dimensions. To ensure content validity, as recommended by Hinkin [33] and Zickar [85], the initial item pool was subjected to pre-testing with a group of 20 participants, including both academics and practitioners. The participants consisted of doctoral-level academics in management and practitioners holding senior or middle management positions in various organizations. The instrument was assessed for comprehensibility and its ability to represent all facets of the construct. Based on the feedback, some items were revised, and ten additional items, suggested by respondents, were incorporated. This resulted in a final pool of thirty-eight items for the SMMWC scale, ensuring comprehensive coverage of the construct.

Table 2 provides a comprehensive list of the total items developed for the SMMWC scale under each dimension, along with explanatory comments on how each item was formulated and derived. This process ensured that the scale captured the desired construct and achieved content validity following established guidelines.

**Table 2:** Proposed items for Social Media Monitoring from Workplace Contacts (SMMWC) scale - Study 1.

Dimensions and Items	Process of Developing Items
**Dimension 1: Perception**	
D1.1My personal social media account is being monitored by my employers and supervisors at least part of the time.	Adapted from surveillance index by Botan (1996).
**D1.2My employers and/or supervisors use social media to “keep tabs” on me**	Adapted from the online obsessive relational intrusion scale by Chaulk and Jones (2011).
D1.3My employers and/or supervisors read my wall conversations (posts and replies).	Adapted from the online obsessive relational intrusion scale by Chaulk and Jones (2011).
D1.4My employers and/or supervisors use my private social media profile to obtain information about me.	Adapted from the online obsessive relational intrusion scale by Chaulk and Jones (2011).
D1.5My employers and/or supervisors use social media to monitor who I talk to/am friends with.	Adapted from the electronic intrusion perpetration scale by Reed et al. (2016).
D1.6My employer and/or supervisor use social media to generally know what are my interests and what I do outside the office.	Developed inductively^*^
D1.7My employer and/or supervisor use social media to cross-check what I say at the workplace.	Developed inductively
**D1.8My personal social media account is being monitored by my colleagues at least part of the time.**	Adapted from surveillance index by Botan (1996).
**D1.9My colleagues use social media to “keep tabs” on me.**	Adapted from the online obsessive relational intrusion scale by Chaulk and Jones (2011).
D1.10My colleagues read my wall conversations (posts and replies).	Adapted from the online obsessive relational intrusion scale by Chaulk and Jones (2011).
D1.11My colleagues use my private social media profile to obtain information about me.	Adapted from the online obsessive relational intrusion scale by Chaulk and Jones (2011).
D1.12My colleagues use social media to monitor who I talk to/am friends with.	Adapted from the electronic intrusion perpetration scale by Reed et al. (2016).
D1.13My colleagues use social media to generally know what are my interests and what I do outside the office.	Developed inductively
D1.14My colleagues use social media to cross-check what I say at the workplace.	Developed inductively
**Dimension 2: Potential** D2.1Most of the information posted on my social media account is visible to my workplace contacts.	Developed theoretically^*^
D2.2I find it difficult to hide information from my workplace contacts on my social media account.	Developed theoretically
D2.3I find it difficult to keep track of who from my workplace contacts is monitoring my social media account.	Developed theoretically
**D2.4The information I post on social networking sites can be used against me at any stage of my career.**	Developed theoretically
**D2.5The settings of my social media account are such that workplace contacts can search extensively about me.**	Developed theoretically
**D2.6The settings of my social media account are such that workplace contacts can analyze and use information from my social media account.**	Developed theoretically
**Dimension 3: Management Policy**	
D3.1My organization has a social media policy in place.	Developed theoretically
D3.2My employer uses the private social media information of employees to ensure that employees adhere to social media policies set by the company.	Developed theoretically
D3.3My employer uses private social media information about employees to make hiring decisions.	Developed theoretically
**D3.4My employer uses private social media information about employees to make promotion decisions.**	Developed theoretically
D3.5My employer uses private social media information about employees to penalize employees.	Developed theoretically
**D3.6My employer uses private social media information about employees to fire employees.**	Developed theoretically
**D3.7My employer expects me to have a social media presence.** **D3.8My employer judges my cultural fit in the organization from my social media activities.**	Developed inductively Developed inductively
**Dimension 4: Maturity** D4.1My organization communicates social media policies and rules to employees (through meetings, seminars, emails, etc.).	Developed inductively
D4.2My organization requires employees to sign a contract adhering to social media policies and rules of the organization.	Developed inductively
D4.3My organization has a department or designated individual(s) to monitor social media news relating to my company.	Developed inductively
**D4.4My organization has summoned employees for an explanation call for violating social media policies or implied social media norms.**	Developed theoretically
**D4.5My organization has taken disciplinary action against employees for violating social media policies or implied social media norms.**	Developed theoretically
**D4.6My organization has taken legal action against employees for violating social media policies or implied social media norms.**	Developed theoretically
**D4.7There is a formal mechanism in place for my colleagues to report employees for violating social media policies or implied social media norms.**	Developed theoretically
D4.8There is an informal mechanism in place for my colleagues to report employees for violating social media policies or implied social media norms.	Developed theoretically
D4.9My colleagues have ostracized (excluded from the social circle) employees for violating social media policies or implied social media norms.	Developed theoretically
D4.10My colleagues have judged employees for violating cultural norms on social media.	Developed inductively

* Items in bold are items retained in the final scale based on factor loadings

* The term “developed theoretically” means that the researcher used Foucault’s (1977) and Botan’s (1996) theoretical frameworks, as a guide for developing items.

* The term “developed inductively” refers to the items suggested by respondents during the content validity stage.

#### Questionnaire administration for study 1.

After item generation and content validity, the next stage involves administering the questionnaire to the target population and conducting psychometric analysis on the new scale to assess its construct validity and reliability [33]. To establish construct validity, a comprehensive questionnaire was administered in Study 1, which included thirty-eight proposed items for the SMMWC (Table 2). The questionnaire also included variables measuring EPM and social media monitoring from the nomological network to test the discriminant validity of the scale. Additionally, variables measuring job satisfaction, invasion of privacy, online disclosure, and identity clarity were included in the questionnaire to test the criterion validity of the scale.

Participants were provided with detailed information regarding the study’s objectives, procedures, confidentiality measures, and their rights. Prior to their involvement in the study, informed written consent was obtained from all research participants. Data management procedures adhered to pertinent data protection regulations.

Measures of Study 1. All items in the measures were anchored to a five-point Likert scale, with 1 referring to “strongly disagree” and 5 referring to “strongly agree”.

SMMWC: SMMWC was measured using the 38-item scale developed deductively and inductively for this study, as explained in the preceding subsection. The items for the scale are presented in Table 2.

EPM: EPM was measured using three items from Wells et al.’s scale [86]. The term: “call monitoring system” was replaced with “EPM devices”. A sample item is “My organization monitors the activities of employees through EPM devices to detect possible misconduct or fraud.”

SMM: Social media monitoring from multiple audiences was measured using three items from Child and Starcher’s “Concerns about Mediated Lurking” scale [59]*.* The word “Facebook” was replaced with “social media.” A sample item is, “I worry about who may be engaging in prolonged scrutiny of my social media page”.

Job Satisfaction: Job satisfaction was measured using three general job satisfaction items from the Job Descriptive Index developed by Hackman and Oldham [87]. A sample item is “Generally speaking, I am very satisfied with this job.”

Invasion of Privacy: Employees’ invasion of privacy was measured using Posey et al.’s five-item privacy invasion scale [62]. The original term “use of the computer system” was omitted from the scale. A sample item is “I feel personally invaded by the methods used by my organization to collect information about its employees.”

Online Self-Disclosure: Employees’ online self-disclosure was measured using a four-item self-disclosure scale on Facebook by Wang and Stefanone [88] after replacing the term “Facebook” with social media. A sample item is “I often talk about my feelings on social media.”

Participants of Study 1. The survey was administered to 334 employees from the banking and IT sectors across Islamabad, Karachi, and Lahore, Pakistan. Using purposive sampling, participants were selected for their active social media usage and the presence of workplace contacts on their social media accounts. The sample included 181 men and 153 women, aged 22 to 45, with diverse educational backgrounds (primarily undergraduate and postgraduate). On average, respondents had five years of work experience, spent four hours daily on social media, and most posted content several times a week.

#### Results of study 1.

Initial Item Reduction. To examine the latent structure of the SMMWC scale in Study 1, a series of exploratory factor analyses were conducted using IBM SPSS Statistics version 25 on the 38 items. Prior to conducting the factor analysis, the data’s suitability was assessed using the Kaiser-Meyer-Olkin (KMO) measure of sampling adequacy and Bartlett’s Test of Sphericity. The KMO value was.819, indicating a high level of sampling adequacy, and Bartlett’s Test of Sphericity was significant (χ²(378) =  3722.324, p < .001), suggesting that the correlations between items were sufficiently large for factor analysis. Principal component analysis with Varimax rotation and Kaiser normalization was used to identify items with strong and distinct loadings on the components, guiding the potential reduction of factors. The final scale resulted in a reduced set of fifteen items loaded on four factors with strong and unique loadings. Items with factor loadings greater than 0.4 were considered for retention [89]. Table 3 shows the factor loadings of SMMWC on the items retained in the scale. Out of the total items, Item 5, originally in the dimension “potential” was cross-loading with items under the dimension of “management policies.” However, it was retained under the dimension of “potential” due to its relevance with the dimension.

**Table 3:** EFA on selected items of SMMWC - Study 1.

SMMWC Component					
	**Previously** **named as**	**1**	**2**	**3**	**4**
1.My personal social media account is being monitored by my employers and supervisors at least part of the time.	D1.1			0.756	
2.My employers and/or supervisors use social media to “keep tabs” on me.	D1.2			0.786	
3.My personal social media account is being monitored by my colleagues at least part of the time.	D1.8			0.705	
4.My colleagues use social media to “keep tabs” on me.	D1.9			0.768	
5.The information I post on social networking sites can be used against me at any stage of my career.	D2.4	0.518			0.466
6.The settings of my social media account are such that workplace contacts can search extensively about me.	D2.5				0.855
7.The settings of my social media account are such that workplace contacts can analyze and use information from my social media account.	D2.6				0.803
8.My employer uses private social media information about employees to make promotion decisions.	D3.4	0.653			
9.My employer uses private social media information about employees to fire employees.	D3.6	0.768			
10.My employer expects me to have a social media presence.	D3.7	0.705			
11.My employer judges my cultural fit in the organization from my social media activities.	D3.8	0.745			
12.My organization has summoned employees for an explanation call for violating social media policies or implied social media norms.	D4.4		0.604		
13.My organization has taken disciplinary action against employees for violating social media policies or implied social media norms.	D4.5		0.834		
14.My organization has taken legal action against employees for violating social media policies or implied social media norms.	D4.6		0.836		
15.There is a formal mechanism in place for my colleagues to report employees for violating social media policies or implied social media norms.	D4.7		0.735		

Extraction Method: Principal Component Analysis. Rotation Method: Varimax with Kaiser Normalization

Values less than 0.4 have been suppressed

After selecting the items for the scale, the “split-halves” method, which is a reasonable alternative to collecting two separate samples of data to perform an EFA and CFA [33] was used to confirm the factor structure of the selected scale. The factor structure was retained at random in 30%, 50%, and 70% of the datasets.

**Fig 1:**
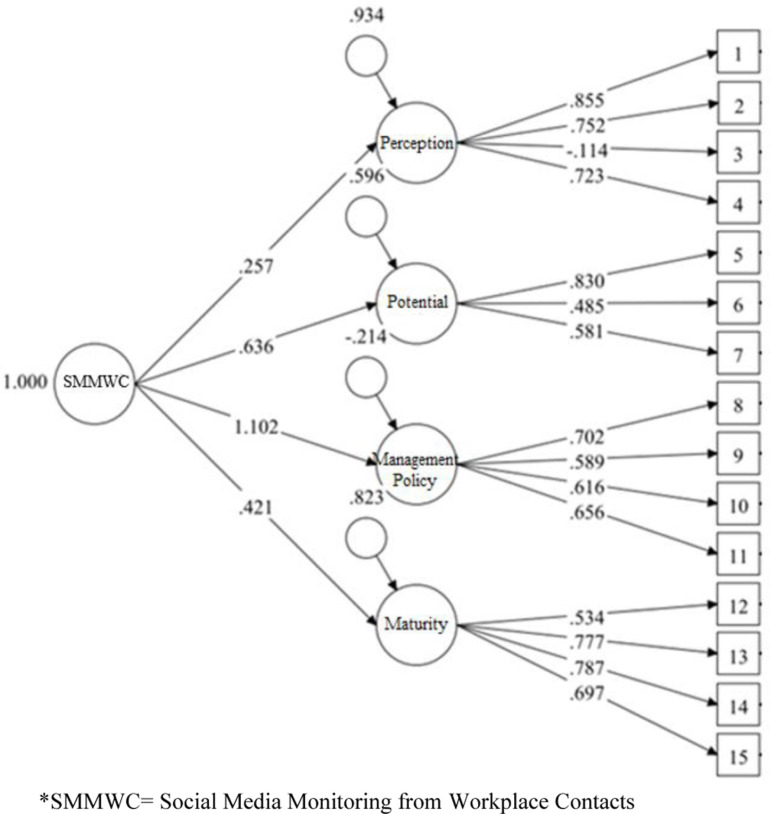
Confirmatory factor analyses for dimensions of SMMWC - Study 1.

Confirmatory Factor Analyses. The four-factor structure of the SMMWC scale was then assessed using CFA on MPlus 8 as a second-order latent construct [90]. Table 4 presents the model fit information for the different models tested. Model fit indices, such as comparative ﬁt index (CFI) and the Tucker-Lewis Index (TLI) were preferred over Goodness-of-Fit Index (GFI) and the Adjusted Goodness-of-Fit Index (AGFI), as these indices consider model complexity and are less affected by sample size, providing a more dependable fit assessment than GFI and AGFI [91]. According to several authors, the general guidelines to evaluate the goodness of ﬁt are that the cut-off value of the CFI and the Tucker-Lewis Index TLI of the model should be closer to.95 and the cut-off value of the mean square error of approximation (RMSEA) and the standardized residual values of the model (SRMR) should be closer to.06 and.08 respectively [91]. The four-factor model showed an adequate fit to the data (Table 4, Study 1, Model 2). All items’ standardized loadings were higher than.45, and the error variances ranged from.364 to.789. The model was further improved after covarying the error variances of some of the indicators in the model to improve fit statistics, a procedure described in many textbooks on structural equation modeling (SEM) (for example, [92,93]. The revised model after covarying the selected error terms (items within the dimension), with modification indices greater than 20 had the following statistics: X2(81) =  1582, p = 0.00; CFI = .917; TLI = .892; RMSEA = .068; SRMR = .005 (Table 4, Study 1, Model 3). The results of the four-factor structure of SMMWC were much better than a single-factor structure model with SMMWC (Table 4, Study 1, Model 1) suggesting that the dimensions of SMMWC represented the model better. Fig 1 shows the CFA results for the SMMWC, illustrating the factor structure and loadings.

**Table 4:** Model fit information - Study 1 and Study 2.

	*X* ^*2*^	df	CFI	TLI	RMSEA	SRMR
**Study 1**						
Model 1 - SMMWC as a single-factor model	899.1	90	.454	.363	.164	.128
Model 2 - SMMWC as a four-factor model without modification Indices	287	86	.864	.835	.084	.063
Model 3 - SMMWC as a four-factor model with modification Indices	1582	81	.917	.892	.068	.005
Model 4 - Items of SMMWC, EPM, and SMM loaded on a single latent variable	1609.2	189	.397	.330	.150	.129
Model 5 - Items of EPM, SMM, and SMMWC loaded as a separate latent variable	563.5	177	.835	.805	0.081	.077
**Study 2**						
Model 1 - SMMWC as a single-factor model	980.9	90	.666	.611	.183	.104
Model 2 - SMMWC as a four-factor model without modification Indices	262.3	86	.934	.919	.083	.060
Model 3 - SMMWC as a four-factor model with modification Indices	159.9	83	.971	.964	.056	.038
Model 4 - Outcomes^*^ as a single-factor model	3430	594	.396	.369	.127	.131
Model 5 - Outcomes as a six-factor model	1728	582	.756	.736	.082	.094
Model 6 - Outcomes as a six-factor model after removing items with factor loading less than 0.5	726	267	.857	.841	.073	.060
Model 7 - Outcomes as a six-factor model with modification indices.	639	358	0.929	0.920	0.052	0.055
Model 8 – Structural Equation Modeling	2810	1767	.890	.882	.045	.067

SMMWC= Social Media Monitoring from Workplace Contacts.

EPM= Electronic Performance Monitoring

SMM= Social Media Monitoring

* Outcomes = Outcomes in Study 2 include Online Disclosure, Social Capital, Invasion of Privacy, Job Satisfaction, Emotional Exhaustion, and Self-Concept Clarity

Discriminant Validity. To assess the discriminant validity of the developed scale [33,94], a confirmatory factor analysis (CFA) was conducted using three variables: EPM, SMM, and SMMWC. The items of EPM and SMM were loaded as a first-order latent construct, while the items of SMMWC were loaded as a second-order latent construct (Table 4, Study 1, Model 5). Additionally, an alternative model was tested where all items of EPM, SMM, and SMMWC were loaded onto a single-factor latent variable. This was done to determine whether all items represented the same construct or if they were indicators of different variables (Table 4, Study 1, Model 4).

**Fig 2:**
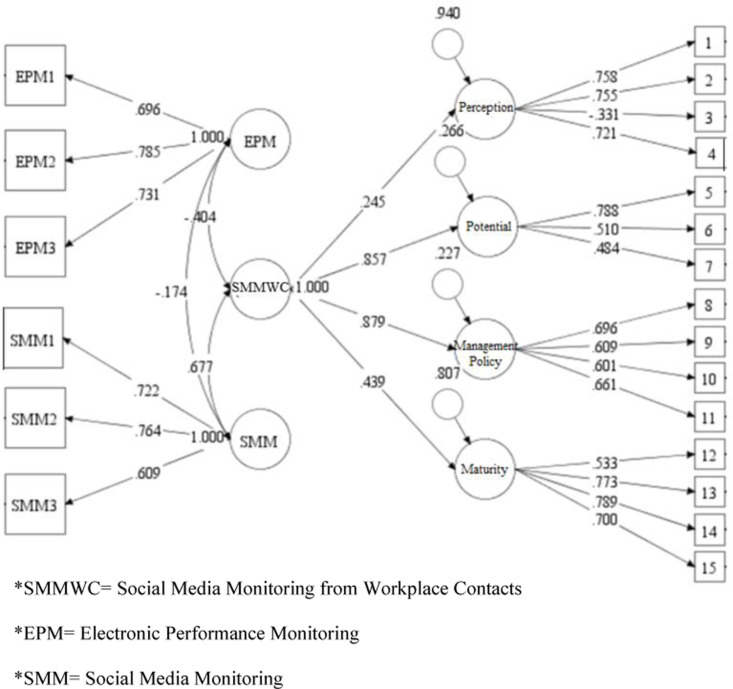
Confirmatory factor analyses for dimensions of SMMWC, EPM, and SMM - Study 1.

The model fit information for the three-variable model (EPM and SMM as first-order latent variables, and SMMWC as a second-order latent variable) yielded better results (X2(177) =  563.5, p = 0.00; CFI = .835; TLI = .805; RMSEA = .081; SRMR = .077) (Table 4, Study 1, Model 5) as compared to the model fit information for the single latent variable model (Table 4, Study 1, Model 4). These results suggest that EPM, SMM, and SMMWC are indeed distinct and discriminant variables (Fig 2).

Criterion-Related Validity. To test the criterion-related validity of the scale, several outcome variables were correlated with the second-order construct of the SMMWC and each of the dimensions of the SMMWC, as presented in Table 5. It was proposed that SMMWC would be positively correlated with employees’ invasion of privacy and negatively correlated with their job satisfaction and online disclosure. The results indicated that the second-order latent construct of SMMWC was positively correlated to employees’ invasion of privacy (0.335 with p < 0.05) supporting the criterion-related validity of the scale. However, SMWWC was not related to job satisfaction in this study. Furthermore, the results indicated that online disclosure was positively correlated with the SMMWC (0.455 p < 0.01) unlike what was hypothesized.

**Table 5:** Correlation between variables of Study 1.

		1a	1b	1c	1d	2	3	4	5	6
**1a**	Perception	1								
**1b**	Potential	.110^*^								
**1c**	Management Policy	.184^**^	.463^**^							
**1d**	Maturity	−0.024	.237^**^	.364^**^						
**2**	SMMWC	.488^**^	.706^**^	.781^**^	.607^**^					
**3**	EPM	.285^**^	−.241^**^	−.313^**^	−0.066	−.133^*^				
**4**	SMM	.161^**^	.402^**^	.366^**^	.326^**^	.487^**^	−.125^*^			
**5**	Invasion of Privacy	.375^**^	.116^*^	.266^**^	.111^*^	.335^**^	0.032	.280^**^		
**6**	Job Satisfaction	−.163^**^	0.096	0.085	.164^**^	0.071	−0.089	0.031	−0.009	
**7**	Online Self Disclosure	−0.044	.361^**^	.317^**^	.543^**^	.455^**^	−0.097	.326^**^	.120^*^	.291^**^

SMMWC= Social Media Monitoring from Workplace Contacts.

EPM= Electronic Performance Monitoring

SMM= Social Media Monitoring *Note:* Asterisks indicate statistical significance levels for p-values, with *** indicating p < .001, ** indicating p < .01, and * indicating p < .05.

### Study 2

#### Questionnaire administration for study 2.

After establishing the construct, criterion, and discriminant validity, Study 2 was conducted to replicate the factor structure of the SMMWC scale and to assess the impact of SMMWC on outcomes. Study 2 was a time-lagged study where data about SMMWC was collected at T1 and data about outcomes (employees’ online disclosure, invasion of privacy, job satisfaction, social capital, emotional exhaustion, and self-concept clarity) was collected at T2, with at least a one-month interval. The questionnaire collection period for Study 2 spanned from 12th July 2022 to 20th February 2023.

Measures of Study 2. All items in the measures used were anchored to a five-point Likert scale with 1 referring to “strongly disagree” and 5 referring to “strongly agree”.

SMMWC (Time 1): SMMWC was measured using a 15-item scale developed in Study 1.

Online Self-Disclosure on Social Media (Time 2): Online self-disclosure of employees was measured using a sixteen-item, social media self-presentation scale by Yang [95]. The scale was divided into four factors breadth, depth, positivity, and authenticity. A sample item is “I display photos that involve a variety of images of me on social media.”

Invasion of Privacy (Time 2): Employees’ invasion of privacy was measured by adapting a 5-item privacy invasion scale by Posey et al. [62] used in Study 1.

Job Satisfaction (Time 2): Job satisfaction was measured using the five-item job satisfaction index by Judge et al., [96]. A sample item is “I feel fairly well satisfied with my present job”.

Emotional exhaustion (Time 2): Emotional exhaustion was measured using five items of emotional exhaustion from the Maslach Burnout Inventory by Maslach and Jackson [79]. A sample item is “I feel mentally drained by my work.”

Self-Concept Clarity (Time 2): Self-concept clarity was measured using the 12-item scale of self-concept clarity scale by Campbell et al [72]. A sample item is “My beliefs about myself often conflict with one another” (reverse coded).

Social Capital (Time 2): Social Capital was measured by using a 10-item online social capital scale by [84] The term “Facebook” was replaced with “social media account” to include additional social media platforms in the data. A sample item for the social capital scale is: “Among the coworkers on my social media account, there are several people I trust to help solve my problems.”

Participants of Study 2. The final data set comprised 302 employees from various industries in Pakistan. Participants were active social media users, had workplace contacts on their social media accounts, and had at least one year of experience in their respective organizations. Informed written consent was obtained from all participants. The survey included 166 men and 136 women, with an average of five years of work experience. Respondents reported spending an average of four hours per day on social media.

#### Results of Study 2.

**Fig 3:**
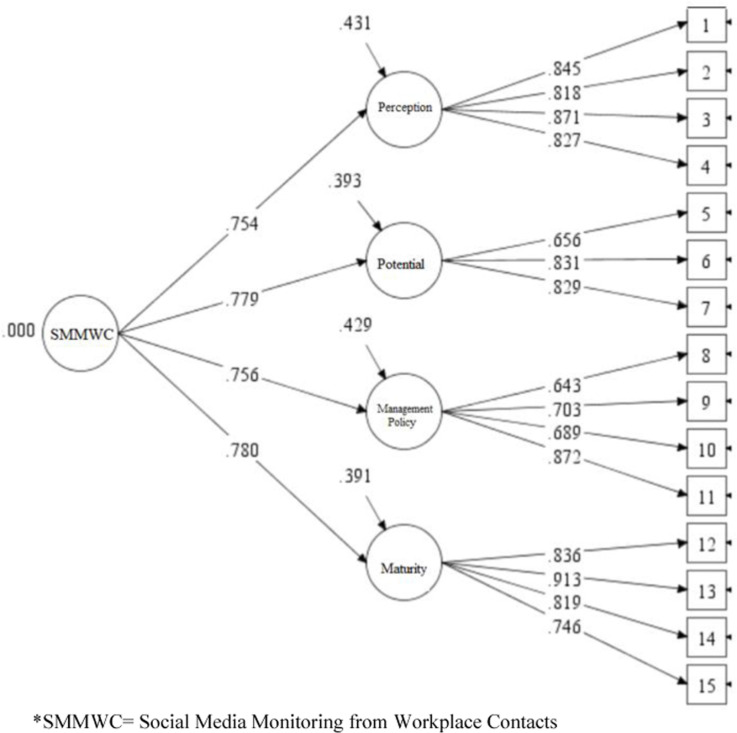
Confirmatory factor analyses for dimensions of SMMWC - Study 2.

Similar to the procedure conducted in Study 1, SMMWC as a single-factor model and SMMWC as a four-factor model were tested in Study 2. The model fit information of the four-factor model of SMMWC showed better results (Table 4, Study 2, Model 2) as compared to the single-factor model (Table 4, Study 2, Model 1). The model was further improved after covarying the selected error terms (items within the dimensions) with modification indices greater than 20: *X*^*2*^(83) =  159, p = 0.00; CFI = .971; TLI = .964; RMSEA = .056; SRMR = .038 (Table 4, Study 2, Model 3). Fig 3 shows the four-factor measurement models at T1.

At T2, data was collected about outcome variables to be tested with SMMWC which included online self-disclosure of employees, invasion of privacy, job satisfaction, social capital, job stress, and self-concept clarity, where social capital consisted of two dimensions, bridging and bonding social capital, making it a third-order latent construct, and other variables, second-order latent constructs. A baseline six-factor model was tested to establish discriminant validity, where the six variables were treated as a unique construct. The values of the six-factor model were below the threshold values for all model indices (Table 4, Study 2, Model 5). However, the model fit indices were still better than the model fit indices of a single-factor model (Table 4, Study 2, Model 4). The factor loadings of all the variables from the six-factor model were analyzed to improve the indices. After analyzing the data, it was found that reverse-coded items in the data had factor loadings of less than 0.5. After removing items with less than 0.5-factor loadings, the model was retested. The revised six-factor model adequately fitted the data (Table 4, Study 2, Model 6). The model further improved after covarying the error variances of some of the indicators in the model. After covarying selected error terms with values greater than 20, the revised model had the following statistics: X2(358) =  639, p = .00; CFI = .929; TLI = .920; RMSEA = .052; SRMR = .055 (Table 4, Study 2, Model 7).

**Fig 4:**
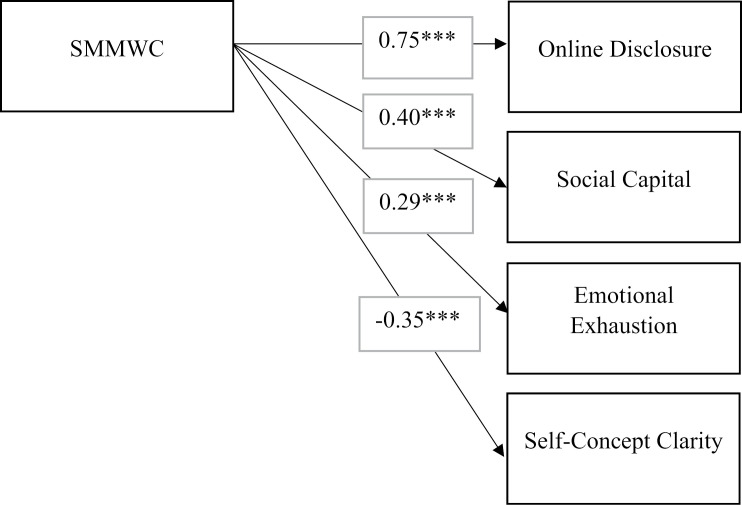
Results of hypothesis testing: Relationship between SMMWC and Outcomes - Study 2.

It was hypothesized that SMMWC will be positively associated with social capital, invasion of privacy, and emotional exhaustion; and negatively associated with online disclosure, job satisfaction, and self-concept clarity. Structural equation modeling was conducted on MPLUS. The results showed a good model fit: χ2(1767) =  2810, p < .05, CFI = .890, TLI = .882, RMSEA = .045, SRMR = .067 (Table 4, Study 2, Model 8). The standardized path coefficients and their significance levels are presented in Table 6. SMMWC had a significant positive effect on online disclosure (β =  0.75***, p < .001), social capital (β =  0.40***, p < .001) and emotional exhaustion (β =  0.29***, p < .001). Additionally, SMMWC had a negative impact on self-concept clarity (β =  -0.35***). However, there was no significant relationship between SMMWC and the outcomes, invasion of privacy (β =  -0.02), and job satisfaction (β =  -0.08). The control, supervisor on the SNS account was positively associated with job satisfaction (β =  0.20**, p < 0.05); the control gender was negatively associated with invasion of privacy (β =  -0.20***, p < 0.05); and the control variable, average hours spent on SNS was associated with the outcome of Emotional exhaustion (β =  0.12 * , p <  0.05). Fig 4 illustrates the results of hypothesis testing, showcasing the relationship between SMMWC and outcomes.

**Table 6:** Regression results of SMMWC on outcomes - Study 2.

			Dependent Variables				
		Online Disclosure	Social Capital	Invasion of Privacy	Job Satisfaction	Emotional Exhaustion	Self-Concept Clarity
	Residual Variances	0.43^***^	0.82^**^	0.96^***^	0.94^***^	0.89^***^	0.87^***^
Controls	Supervisor added	0.07	0.10	0.00	0.20^**^	−0.11	−0.01
	Gender	0.05	0.04	−0.20^***^	0.11	.06	−0.00
	Hrs. spent on SNS	0.05	0.02	0.04	0.01	0.12^*^	−0.10
SMMWC	SMMWC	0.75^***^	0.40^***^	−0.02	−0.08	0.29^***^	−0.35^***^

*Note:* Asterisks indicate statistical significance levels for p-values, with

*** indicating p < .001,

** indicating p < .01, and

* indicating p < .05.

## Discussions

This study contributes to the literature on online information-seeking, social media monitoring, and employee surveillance and control by developing a scale to measure SMMWC. The SMMWC scale offers significant advancement over existing measures of social media monitoring, which either focus on a different audience or, if focused on employees, primarily emphasize employer activities like recruitment and screening. Unlike existing measures, the SMMWC encompasses broader factors such as colleague monitoring, organizational social media practices and policies, and privacy settings. This comprehensive approach provides deeper insights into how social media monitoring affects employees and organizational outcomes. By capturing these additional dimensions, the SMMWC scale fills a critical gap in the literature and offers a unique, valuable tool for understanding social media’s role in the workplace.

The validity of the SMMWC scale is demonstrated through its robust internal consistency, strong alignment with theoretical constructs, and excellent empirical fit. Grounded in Foucault’s concept of the panopticon [34] and Botan’s framework for the panoptic effect [35], the scale’s four dimensions effectively reflect these foundational theories. Confirmatory Factor Analysis (CFA) in Study 1 supported the discriminant validity of the scale confirming that SMMWC is unique from related constructs such as EPM and SMM. Further CFA conducted in both Study 1 and Study 2 reinforced the scale’s reliability and validity, highlighting its utility as a novel and comprehensive measure of workplace related social media monitoring perceptions.

While the primary goal of this study was to design and validate the SMMWC scale, its application in examining relationships with employee outcomes revealed valuable insights. For instance, the absence of a relationship between SMMWC and job satisfaction across studies and mixed findings regarding privacy invasion and SMMWC contradicts previous findings by Stoughton et al. [64] and Suen [32]. These results suggest that employees perceive SNS use for screening and monitoring as more invasive compared to the broader SMMWC construct, which captures perceptions of monitoring from all workplace contacts, including employers and colleagues. Similarly, the unexpected positive relationship between SMMWC and online disclosure underscores the utility of the SMMWC scale in advancing theories like the privacy paradox. Despite the concern for privacy from workplace contacts on SNS, users are likely to disclose information online [97]. Additionally, the positive relationship between SMMWC and emotional exhaustion suggests that employees experience stress when they perceive workplace social media monitoring, which may stem from factors such as perceived privacy invasion [98], blurring of work and personal life [14], and pressure to conform on social media. Conversely, the inverse relationship between SMMWC and self-concept clarity, aligns with the fragmentation hypothesis [73–75], indicating that when workers perceive their workplace contacts monitor their social media, they may encounter confusion or uncertainty regarding their self-concept as the pressure of being monitored may enable them to construct multiple identities online, hindering the development of their cohesive self-identity.

Based on these findings, it is recommended that employers and workplace contacts reassess their social media monitoring practices and social media policies to ensure employees’ privacy rights are respected without negatively affecting organizational outcomes. Employees should be provided with workshops and training sessions that educate them on best practices for managing privacy settings and protecting personal information on social media platforms. Organizations should also offer resources such as counseling services and stress management workshops to support employees who may feel stressed or emotionally exhausted and those struggling with self-concept clarity at work. Considering that SMMWC positively impacts social capital, organizations can encourage the bonding of employees over social media if the employees themselves are comfortable, ensuring their privacy and autonomy are respected. These strategies not only mitigate potential negative impacts of SMMWC but also promote a more ethical and supportive workplace environment, addressing both individual well-being and broader societal concerns about privacy and surveillance.

## Limitations and future directions

While the study provides evidence of the psychometric properties of the SMMWC scale, it is important to acknowledge the potential sample biases that may affect the generalizability of the findings. The focus on employees from different industrial sectors in Pakistan could limit the generalizability to other regions and industries. Additionally, the sample’s emphasis on active social media users with workplace contacts may overlook the perspectives of less digitally engaged employees. Future studies are required to test and retest the reliability and construct validity of the scale in different contexts to understand whether its psychometric properties will hold in different national contexts and cultures. It is advisable to retest the full scale to check whether the four-factor model with the same items is retained in different contexts. Furthermore, while the study found relationships between SMMWC and some of the outcomes, more research is needed to confirm the relationships. Future researchers can also tap into additional employee-and-organization-related outcomes using this scale. Possible outcomes include employee relationships with colleagues, employee trust in management, employee turnover, organizational commitment, organizational performance, and employer brand image. Further studies are needed to determine how SMMWC outcomes differ among individuals according to gender, age, and personality traits. In summary, the 15-item SMMWC scale within four dimensions has a well-defined theoretical basis and promising psychometric support for measuring the perception of social media monitoring from workplace contacts. By creating a measurement scale for the phenomenon of SMMWC, this study has provided a starting point to understand the phenomena that have the potential to impact multiple employee-and-organization-related outcomes.
